# Supplementary material for: Discovery of Oxyacanthine Dihydrochloride Monohydrate Polymorphs from Obfuscated Samples by Microcrystal Electron Diffraction
Source: ChemMedChem. 2025 Dec 12;21(1):e202500601. doi: 10.1002/cmdc.202500601 (PMC12812010; doi:10.1002/cmdc.202500601)
Supplement: Supplementary file 1 — Supplementary Material [file CMDC-21-e202500601-s001.pdf]

Supporting Information

Discovery of oxyacanthine dihydrochloride monohydrate polymorphs from obfuscated samples by MicroED

Jieye Lin[1,6], Orel Paz[1,2,3,6], Johan Unge[4] and Tamir Gonen[1,2,3,5]*

[1] Department of Biological Chemistry, University of California, Los Angeles, 615 Charles E. Young Drive South, Los Angeles, California 90095, United States

[2] Molecular Biology Institute, University of California, Los Angeles, Los Angeles, CA 90095, USA.

[3] Howard Hughes Medical Institute, University of California, Los Angeles, Los Angeles, California 90095, United States

[4] Department of Chemistry, Umeå University, 901 87 Umeå, Sweden

[5] Department of Physiology, University of California, Los Angeles, 615 Charles E. Young Drive South, Los Angeles, California 90095, United States

[6] These authors contributed equally to this work.

[*] Corresponding Author T.G. tgonen@g.ucla.edu

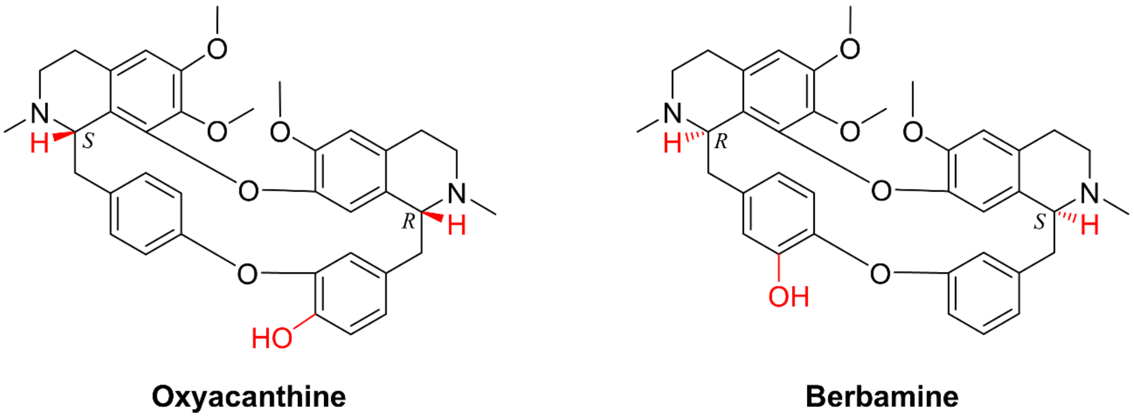

Figure S1 Chemical structures of oxyacanthine and berbamine showing differences in chiral centers and phenylhydroxyl group.

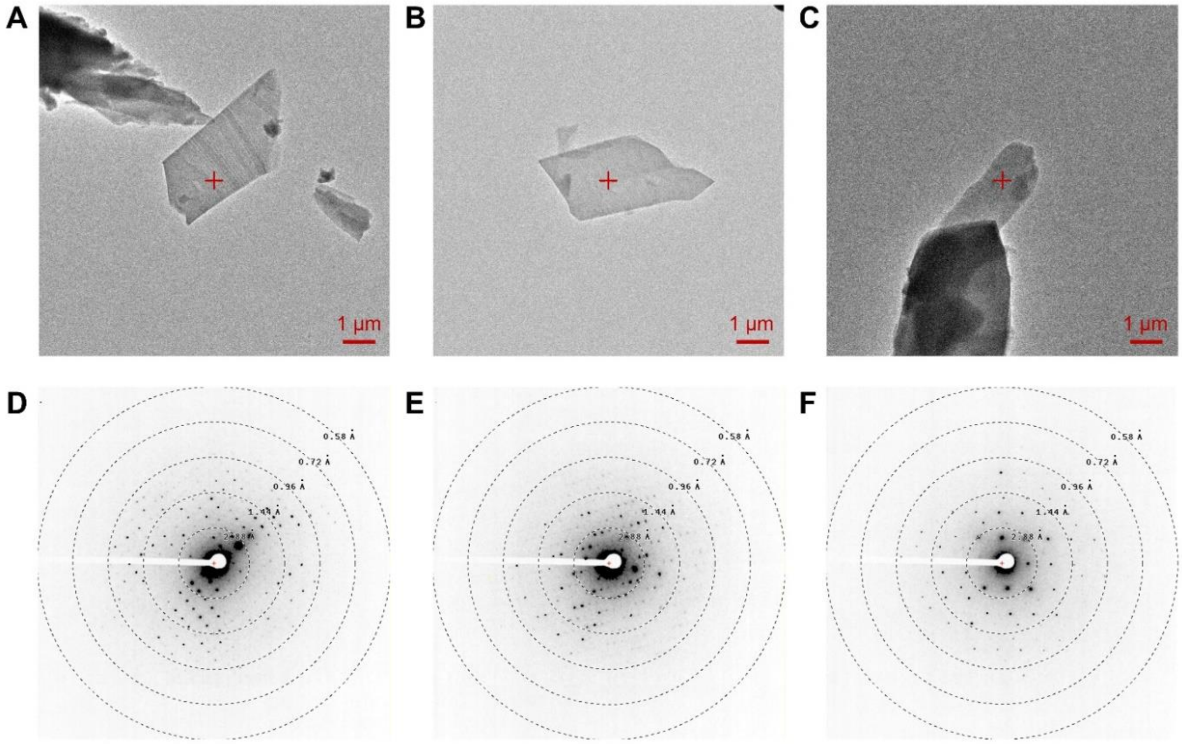

Figure S2 Crystal appearance and diffraction pattern under the TEM. (A-B) Images of **1A** and **1B** (vendor 1) under the imaging mode (SA 5300×). (C) Image of **1B** (vendor 2) under the imaging mode (SA 5300×). (D-E) Diffraction pattern of **1A** and **1B** (vendor 1) under diffraction mode (659 mm). (F) Diffraction pattern of **1B** (vendor 2) under diffraction mode (659 mm).

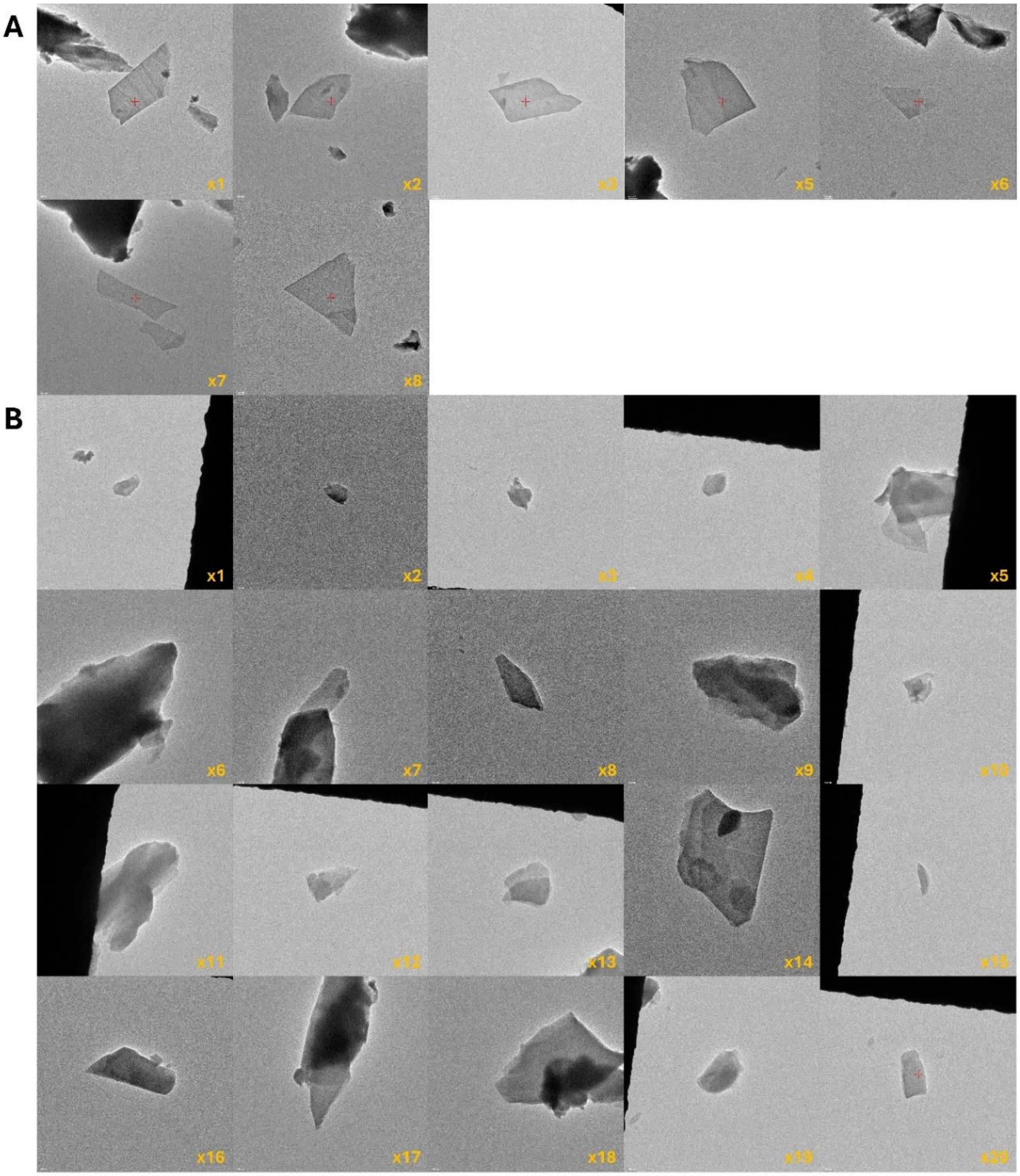

Figure S3 Crystal appearances of “berbamine dihydrochloride” samples obtained from vendors 1 (A) and 2 (B).

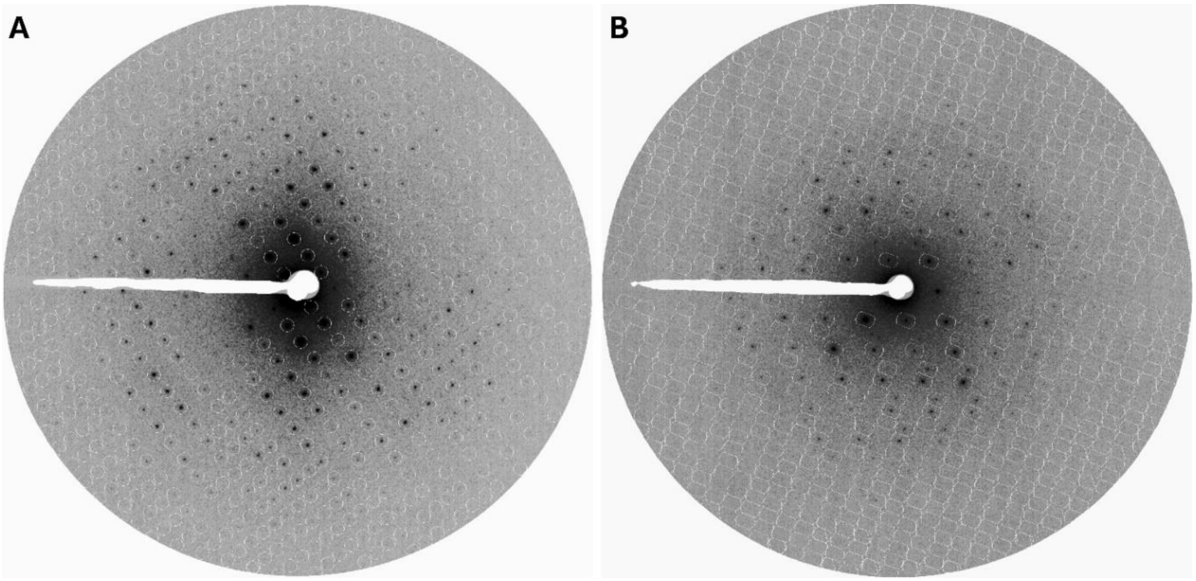

Figure S4 Comparison of the picked and predicted spots suggesting satisfactory index and integration for (A) **1A** and (B) **1B**.

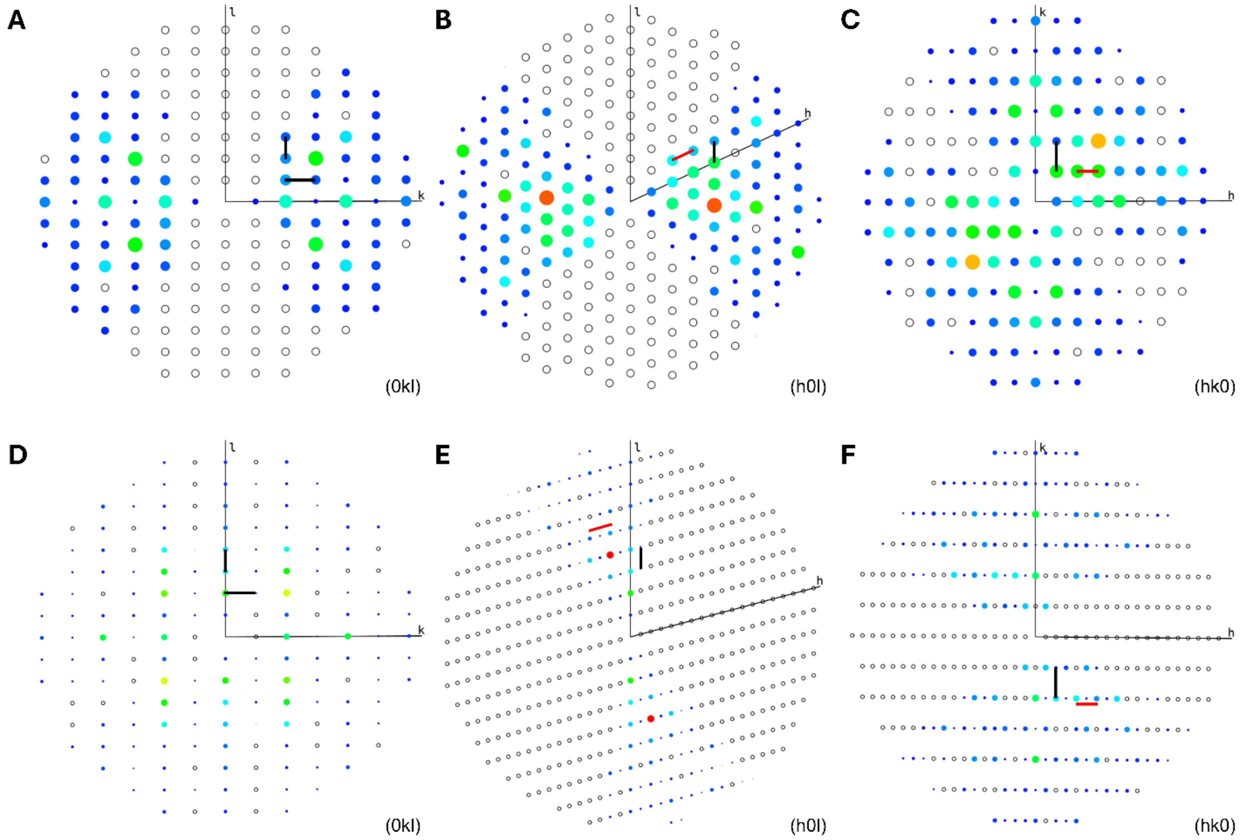

Figure S5 Reciprocal space of (A-C) **1A** and (D-F) **1B**. Reflections were integrated with P1 space group and the resolution was cut at 1.5 Å.

A

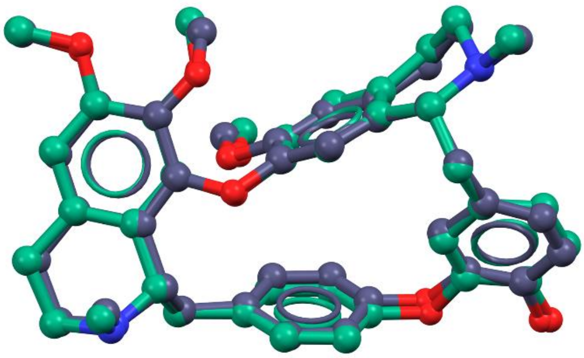

RMSD: 0.304 Å

B

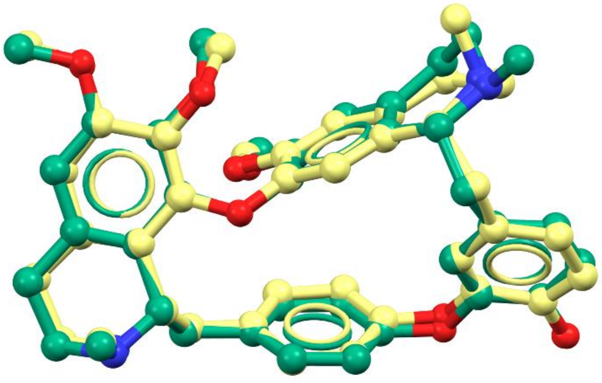

RMSD: 0.469 Å

Figure S6 Superposition of (A) **1A** and (B) **1B** to the literature-reported oxyacanthine (free base) structure.[1] Cl^- ions, water and hydrogen atoms were omitted for clarification. **1A** is colored in purple, **1B** is colored in yellow, the literature-reported oxyacanthine (free base) structure is colored in green.

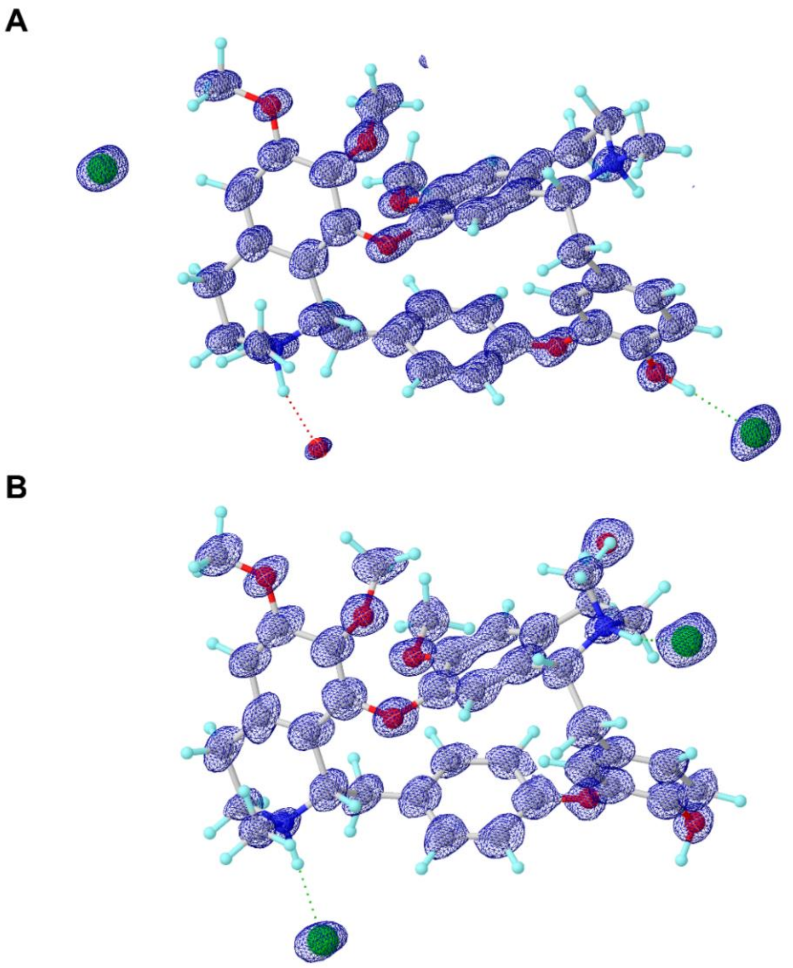

Figure S7 2Fo-Fc maps of **1A** and **1B** (contour level: 3σ)

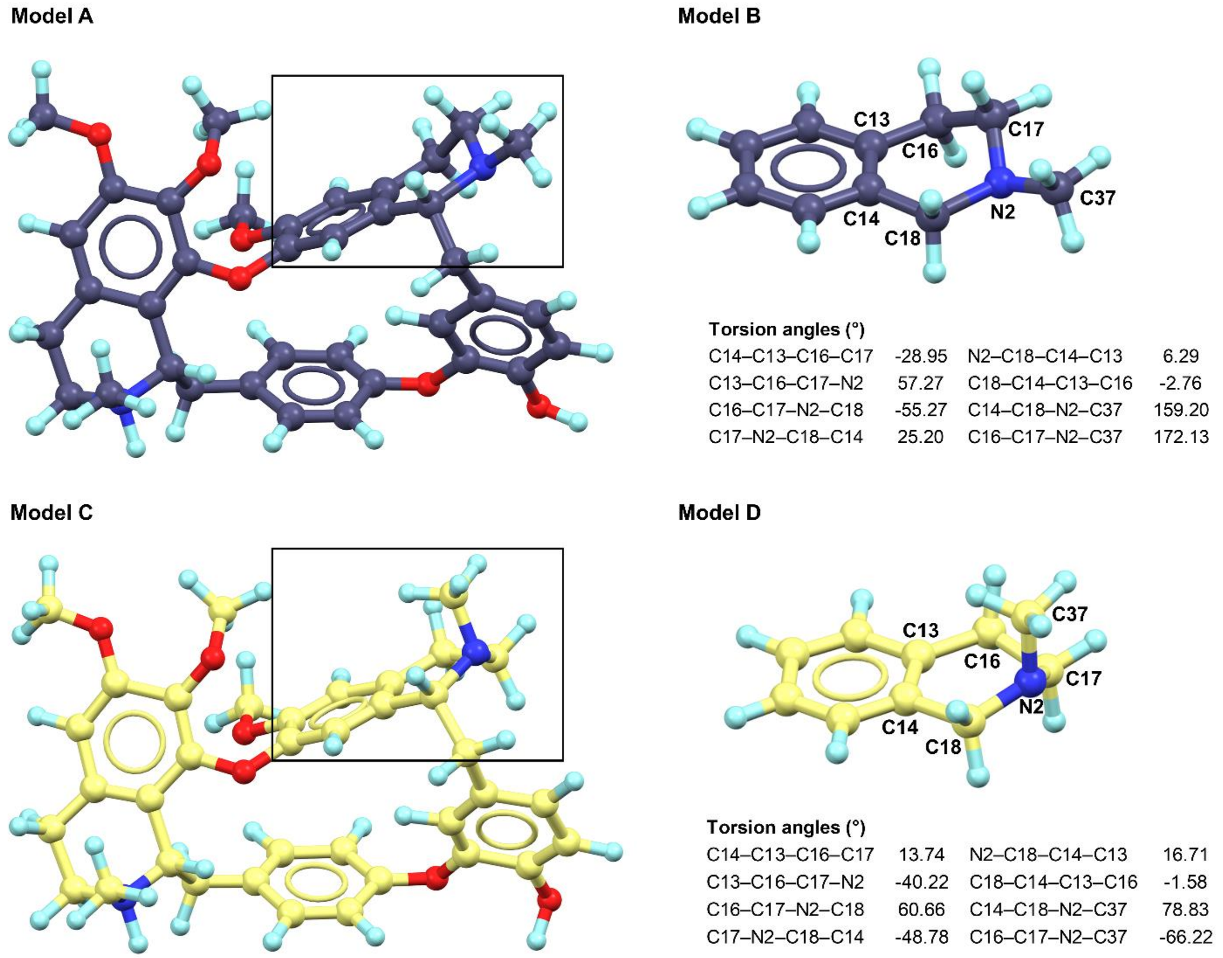

Figure S8 *In silica* models used in DFT calculations. Models A-D were built using the coordinates from **1A** and **1B**.

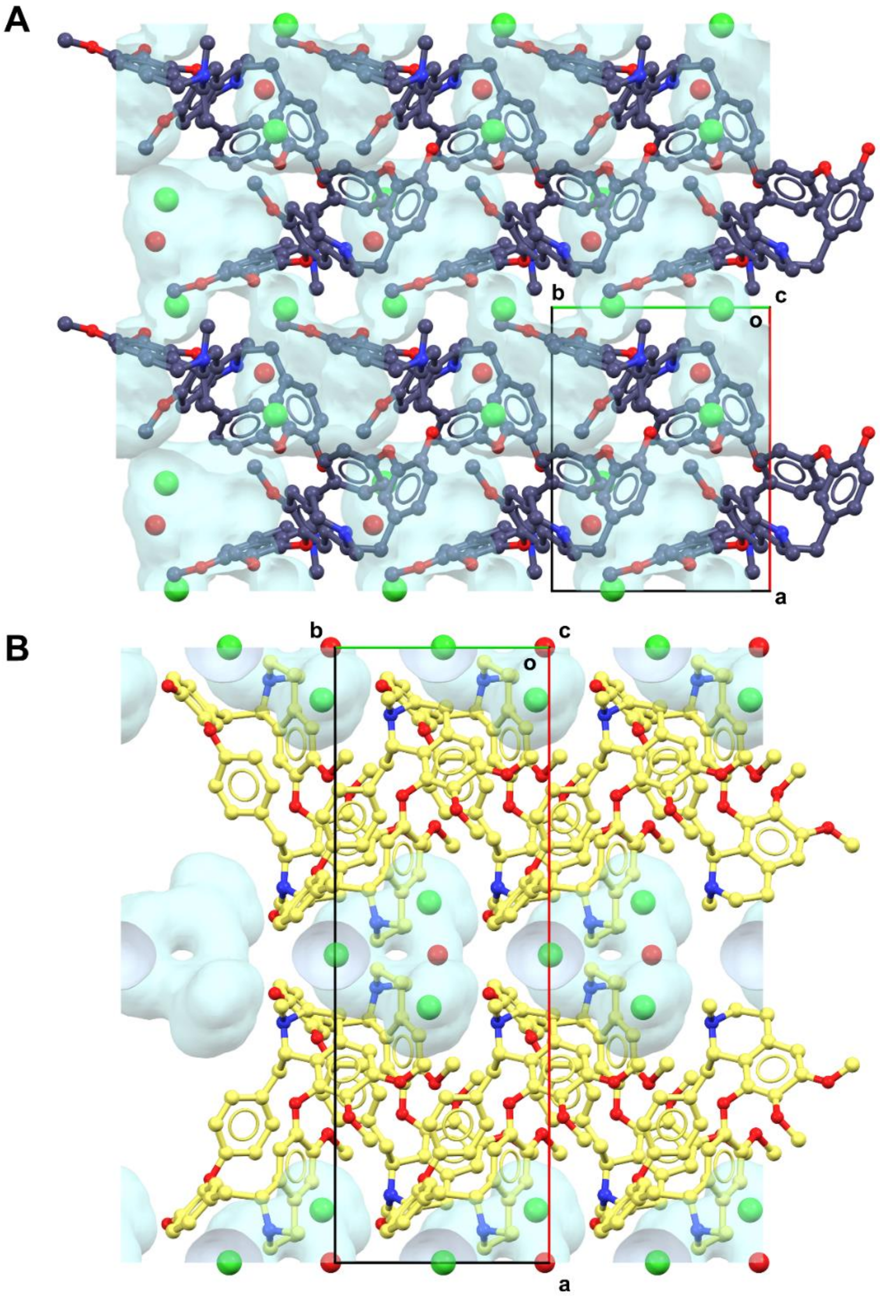

Figure S9 Contact surface (voids) detected in (A) **1A** and (B) **1B** after removal of Cl^{-} ions and water. Probe radius is 1.2 Å with 0.3 Å approximately grid spacing.

Table S1 Statistical analysis of the unit cell parameters (Å, °) indexed from vendors 1 and 2.

Sample	xtal (#)	Space group	**a**	**b**	**c**	α	β	γ
vendor 1	1	$P2_1$	13.60	9.47	14.65	90	115.051	90
	2	$P2_1$	13.57	9.43	14.64	90	114.642	90
	3	C2	27.94	9.31	13.52	90	104.531	90
	4	C2	27.93	9.30	13.59	90	105.316	90
	5	C2	27.90	9.35	13.54	90	104.787	90
	6	$P2_1$	13.64	9.44	14.85	90	115.439	90
	7	$P2_1$	13.61	9.49	14.57	90	115.180	90
	8	$P2_1$	13.70	9.41	15.27	90	114.043	90
	9	C2	27.62	9.37	13.62	90	105.049	90
Sample	xtal (#)	Space group	**a**	**b**	**c**	α	β	γ
vendor 2	1	C2	27.85	9.22	13.67	90	105.327	90
	2	C2	27.81	9.28	13.59	90	105.630	90
	3	P1	7.06	7.42	7.56	115.134	107.137	100.337
	4	C2	27.82	9.30	13.57	90	105.664	90
	5	C2	27.75	9.30	13.54	90	105.493	90
	6	C2	27.68	9.28	13.64	90	105.094	90
	7	C2	27.7	9.29	13.56	90	105.437	90
	8	C2	27.63	9.26	13.60	90	105.264	90
	9	C2	27.73	9.28	13.61	90	105.556	90
	10	C2	28.18	9.27	13.56	90	105.744	90
	11	C2	27.61	9.29	13.69	90	105.432	90
	12	C2	27.75	9.29	13.55	90	105.375	90
	13	C2	27.70	9.28	13.55	90	105.084	90
	14	C2	27.49	9.39	13.56	90	104.617	90
	15	C2	27.74	9.25	13.62	90	105.863	90
	16	C2	27.49	9.25	13.65	90	105.400	90
	17	C2	27.68	9.26	13.62	90	105.638	90
	18	C2	27.55	9.26	13.61	90	104.998	90
	19	C2	27.89	9.28	13.56	90	105.24	90
	20	C2	27.92	9.24	13.59	90	105.398	90

Notes: The vendor 2 xtal 3 is an impurity because the cell volume is too small for oxyacanthine or berbamine.

Table S2 MicroED data statistics of **1A** and **1B**.

	**1A**	**1B**
CCDC deposition number	2469777	2469778
Chemical formula	$C_{37}H_{44}Cl_2N_2O_6$	$C_{37}H_{44}Cl_2N_2O_6$
Molar mass	697.63	697.63
Temperature (K)	80	80
Crystal system	Monoclinic	Monoclinic
Space group	$P2_1$	C2
Unit cell lengths (Å)		
**a**	13.600	27.700
**b**	9.470	9.290
**c**	14.660	13.560
Unit cell angles (°)		
α	90.00	90.00
β	115.05	105.44
γ	90.00	90.00
Cell volume (Å^3)	1710.5	3363.5
No. of merged datasets	1	2
No. of observed reflections	8735	11121
No. of unique reflections	3027	2826
R_{obs} (%)	13.2	18.6
R_{meas} (%)	16.4	21.5
I/Sigma	5.42	5.69
$CC_{1/2}$	98.2	98.8
**Resolution (Å)**	**0.80**	**0.86**
**Completeness (%)**	**81.2**	**95.6**
**R_1 (%)**	**19.28**	**17.19**
wR_2 (%)	37.88	37.34
GooF	1.185	1.161

Table S3 Hydrogen bonding interactions of **1A** and **1B** (Å, °).

**1A**	**D–H**	**H···A**	**D···A**	**D–H···A**
N1–H···OW	1.118	1.740	2.774	151.39
N2–H···Cl2	1.122	2.304	3.176	132.92
O5–H···Cl2	1.000	1.994	2.972	165.40
OW–H···Cl1	–	–	2.932	–
OW–H···Cl2	–	–	3.201	–
**1B**	**D–H**	**H···A**	**D···A**	**D–H···A**
N1–H···Cl1	1.124	2.074	3.065	145.28
N2–H···Cl2	1.123	1.943	3.034	162.97
O5–H···O6	1.001	2.728	2.671	76.16
OW–H···O5	–	–	2.801	–

Reference:

1. Y. Cheng, D. Akramov, L. Yakhshilikova, C. Zhu, J. Lu, J. Suo, S. Pugazh, H. Qin, S. Abduahadi, J. Qin, *J. Nat. Prod.* 2025, **88**, 191.
